# The association between patients’ personality traits and pain perception during orthodontic treatment: a systematic review

**DOI:** 10.3389/fneur.2024.1469992

**Published:** 2024-11-27

**Authors:** Monika Lorek, Anna Jarząbek, Magdalena Sycińska-Dziarnowska, Sylwia Gołąb, Konrad Krawczyk, Gianrico Spagnuolo, Krzysztof Woźniak, Liliana Szyszka-Sommerfeld

**Affiliations:** ^1^Private Clinic HaLo Ortho, Gniezno, Poland; ^2^Laboratory of Paediatric Dentistry, Pomeranian Medical University in Szczecin, Szczecin, Poland; ^3^Department of Maxillofacial Orthopaedics and Orthodontics, Pomeranian Medical University in Szczecin, Szczecin, Poland; ^4^Department of Economics and Accounting, West Pomeranian University of Technology in Szczecin, Szczecin, Poland; ^5^University Dental Clinic, Pomeranian Medical University in Szczecin, Szczecin, Poland; ^6^Department of Neurosciences, Reproductive and Odontostomatological Sciences, University of Naples “Federico II”, Napoli, Italy; ^7^School of Dentistry, College of Dental Medicine, Kaohsiung Medical University, Kaohsiung, Taiwan; ^8^Laboratory for Propaedeutics of Orthodontics and Facial Congenital Defects, Pomeranian Medical University in Szczecin, Szczecin, Poland

**Keywords:** orofacial pain, orthodontic pain, pain perception, personality traits, stomatognathic system

## Abstract

**Background:**

Orthodontic treatment is frequently correlated with different levels of discomfort and pain, caused by the application of forces to move the teeth. The mechanism of orthodontic pain is based on the initially activation of sensory receptors in periodontal tissues which results in a cascade of nociceptive pain processing and transduction in both the central and peripheral nervous systems that is finally experienced by patients. The perception of pain is subjective and varies among people, as it is influenced by both general and individual elements. This systematic review aims to synthesize existing knowledge on the association between patients’ personality traits and pain perception during orthodontic treatment, and its influence on the success of orthodontic therapy.

**Methods:**

The search strategy included the databases PubMed, Scopus, Embase, and Web of Science. The inclusion criteria were studies examining the correlation between personality traits and pain perception in patients undergoing orthodontic treatment. The quality of the studies was assessed using the Newcastle–Ottawa Scale (NOS).

**Results:**

The search strategy yielded 301 potential articles, with 10 papers meeting the inclusion criteria. Five studies were judged at a low risk of bias and another five studies were assessed as having a moderate risk of bias. Most of the studies reported relationship between personality traits and pain perception during orthodontic treatment, as well as treatment attitudes, and post-treatment satisfaction.

**Conclusion:**

Patients’ psychological characteristics seems to affect pain perception and other factors associated with orthodontic treatment. Given that several studies were judged to have a moderate risk of bias, as well as high heterogeneity among studies, further research is needed.

**Systematic review registration:**

The systematic review was registered in PROSPERO database (CRD42024537185).

## 1 Introduction

According to the revised International Association for the Study of Pain (IASP) definition of pain, pain is defined as “an unpleasant sensory and emotional experience associated with, or resembling that associated with, actual or potential tissue damage” (1). Orthodontic treatment is frequently correlated with different levels of discomfort and pain, caused by the application of forces to move the teeth (2). The mechanism of orthodontic pain is based on the initially activation of sensory receptors in periodontal tissues which results in a cascade of nociceptive pain processing and transduction in both the central and peripheral nervous systems that is finally experienced by patients. Approximately 72 to 100% of orthodontic patients report experiencing pain (3–6). The perception of pain is subjective and varies among people, as it is influenced by both general and individual elements. These includes factors such as age, gender, individual pain threshold, emotional or cognitive aspects, degree of stress, physical activity levels, cultural and genetic factors, previous negative dental experiences, as well as the intensity of the applied orthodontic force (7–14). One recent systematic review found that the perception of pain during orthodontic treatment with surgical acceleration intervention was greater in the first 24 h compared to conventional orthodontic treatment, but it was similar after 7 days (15). In addition, a positive correlation between dental anxiety and patient’s pain perception during orthodontic treatment has been observed (16). While the majority of patients finds pain manageable, about 10% of patients experience high intensity leading to the decision to discontinue treatment. The experience of discomfort may substantially decrease the level of cooperation of the patient, and as a consequence may negatively impact on treatment outcome quality, as well as patients’ satisfaction (13, 17–20). Probably due to difficulties in chewing or speech impairment, it may discourage patients from taking care of proper dental treatment (5).

Cooperation between the clinician and the patient, or parents in the case of younger patients, is essential for a positive orthodontic treatment. The patient’s cooperation is assessed based on their consistency in keeping appointments, wearing elastics, headgear, or removable appliances, maintaining proper oral hygiene, and abstaining from chewing hard substances that may distort archwires. It has been proved that some personality traits of patients and their parents, such as, e.g., the emotionality of the child and a sense of self-efficacy and conscientiousness of the parents may significantly impact on the cooperation during orthodontic treatment with removable appliances (21). This is particularly important in the context of treatment with clear removable aligners. It has been suggested that increased levels of neuroticism were more often seen in non-compliant patients during Invisalign treatment (22). It was highlighted that orthodontists do not have a proper ability to treat pain (23), and a fundamental lack of communication between the doctor and the patients leads to difficulties in assessing and predicting the level of pain (9, 24). If patients are more informed about the procedures, they need less pain medication, and a good understanding of the procedure often promotes a positive outcome. Specifically, it was shown that those patients who had previously undergone orthodontic therapy and had more knowledge experienced less pain during treatment (25).

Personality traits are widespread patterns of thinking and behaving that can influence behavior, interests, and satisfaction. Scientifically, they are organized according to the Five Factor Model including Neuroticism (e.g., emotional instability, anxiety and pessimism), Extraversion (e.g., sociability and assertiveness), Openness (e.g., intellect and curiosity), Agreeableness (e.g., compassion and civility), and Conscientiousness (e.g., responsibility and achievement) (26). It has been demonstrated that self-esteem is correlated with each of the Big Five factors (27). Research has shown varying results on the relationship between patients’ personality traits and their attitudes toward orthodontic treatment or their perception of pain (19, 28–31). A significant correlation emerged, linking pain perception with one’s attitude, thereby underscoring its pivotal role as a key factor contributing to treatment discontinuation (23). Furthermore, personality characteristics, such as neuroticism and meticulousness, affected the perception of pain. Generally, patients with better approach experienced less pain, and those with less pain tended to have better approach. Some research indicated that personality traits could influence motivation-for example, psychological disorders might lead to missed appointments. It has also been suggested that a patient’s choice of orthodontic appliance might reflect their personality traits or psychological status, affecting their adaptation and adjustment to the appliances (32). However, other studies argued that a patient’s personality traits do not reliably predict their pain perception or attitude toward orthodontic treatment and level of cooperation (19, 28, 33).

Given the divergence in focus among existing studies regarding the association between patients’ personality traits, pain perception, and their attitude toward orthodontic treatment, there is a need for a comprehensive synthesis of this knowledge. In addition, it should be emphasized that, until now there have been no reviews on the impact of personality traits on pain sensation in patients receiving orthodontic treatment. Hence, the primary goal of this paper is to gather and analyze the existing data from the literature on the interaction between patients’ personality factors and pain perception during orthodontic treatment, and its influence on the success of orthodontic therapy. This study aims to enhance our understanding of how a patient’s personality traits relate to their pain experience during treatment, leading to greater patient fulfillment and satisfaction, and ultimately, improved oral health.

## 2 Methods

The review protocol was officially registered with the PROSPERO International Prospective Register of Systematic Reviews under the registration number CRD42024537185. This review was carried out following the guidelines contained in “Preferred Reporting Items for Systematic Reviews and Meta-Analyses” (PRISMA) (34) (Supplementary Tables S1, S2).

### 2.1 Review strategy

Following the PICOS framework (35), the structure of the systematic review was organized as follows:

Population (P): individuals over the age of 12 who have undergone orthodontic treatment. There were no restrictions concerning the type and severity of malocclusion, orthodontic technique, or the types of orthodontic appliances utilized.

Intervention (I): personality traits. No restrictions were applied with regard to the type of personality traits and the type of tools/tests evaluating patient’s personality traits in the study.

Comparison (C): not applicable.

Outcomes (O): pain perception. No restrictions were applied with regard to the type of tools/tests evaluating patient’s pain sensation in the study.

Study design (S): observational studies (cross-sectional and longitudinal) on the association between patients’ personality factors and pain perception during orthodontic treatment.

**Figure 1:**
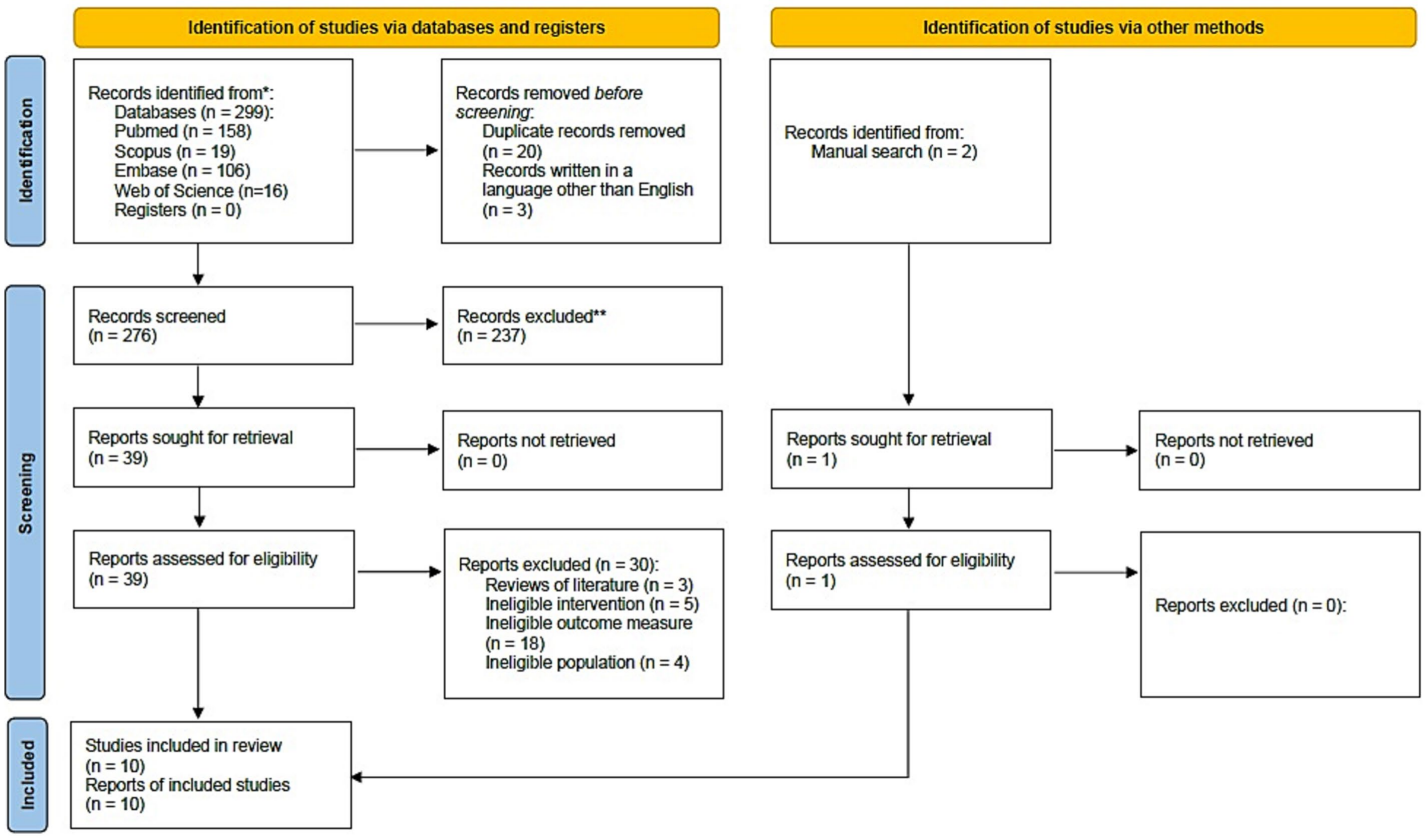
PRISMA flow diagram for the search strategy.

The PICOS question guiding this systematic review was formulated as: “Is there a relationship between personality traits and pain perception among patients receiving orthodontic treatment?” Four databases (PubMed, Scopus, Embase, and Web of Science) were searched electronically by two independent reviewers (M.L. and A.J.) without any limitations on publication dates and using the following keywords: “pain perception” AND “orthodontic” AND “personality.” All relevant publications in English language were examined, in an unbiased manner. The last search was conducted on January 31, 2024, ensuring that all available literature was considered. Additionally, references from relevant articles were manually collected, and a thorough search of the related literature was performed. This search was repeated just before the final analysis to ensure its comprehensiveness (Figure 1).

### 2.2 Eligibility criteria

The following inclusion criteria were applied for this review:

- Study type: Observational studies examining the relationship between patients’ personality traits and pain perception during orthodontic treatment.
- Outcome of interest: Assessments of personality factors and patient-specific characteristics, including their experiences of pain and attitudes toward orthodontic treatment. Analysis of the relationship between personality traits and pain perception in patients undergoing orthodontic treatment, and its influence on the success of orthodontic therapy.
- Object of the study: Exploration of how patients’ personality traits influence pain perception during orthodontic treatment.
- Participants: orthodontically treated human subjects over 12 years of age.

The exclusion criteria for this study were as follows: studies with an ineligible design, such as case reports, reviews, animal studies, unpublished data, or studies not written in English; studies with an ineligible intervention or outcome measure, such as those lacking proper tools or psychological tests to evaluate patients’ personality traits, or missing assessments for pain sensation, and studies focused on surgical treatments; and studies with an ineligible population, including those involving patients with craniofacial congenital anomalies or children under 12 years of age.

### 2.3 Extraction of data

After removing duplicates and papers not written in English, the titles and abstracts of the remaining studies were initially reviewed by the first author (M.L.) and then evaluated by the second author (A.J.) to pinpoint potentially eligible studies. Following this, the full texts of selected papers were thoroughly examined based on preset inclusion and exclusion criteria. Only studies examining the link between personality traits and pain perception in patients undergoing orthodontic treatment, were considered for inclusion. Any ambiguities or uncertainties during the review were resolved by the third author (L.S.-S.). Throughout this process, essential details from the included studies, such as study design, participant characteristics, and outcome measures, including measurement tools, procedures, and data analysis—were systematically collected. The author conducting the final review (M.L.) compiled these results in an Excel spreadsheet. If information was missing, the corresponding authors of the studies were contacted to provide additional details where possible.

### 2.4 Quality assessment

The Newcastle-Ottawa Quality Assessment Scale (NOS) was utilized to assess the quality of non-randomized studies included in the review (36). This evaluation was based on a star system, where stars are assigned to three specific criteria: selection (up to four stars [****]), comparability (up to two stars [**]), and outcome (up to three stars [***]), totaling a maximum of nine stars possible. The overall score determined the risk of bias, categorized as “high risk of bias” (0–3 stars), “moderate risk of bias” (4–6 stars), and “low risk of bias” (7–9 stars). Two authors (M.L. and A.J.) independently performed these quality assessments, and any uncertainties were resolved by a third author (L.S.-S.).

### 2.5 Data synthesis

A PRISMA diagram was created to visually depict the search strategy and the subsequent screening and inclusion processes (Figure 1). After a detailed examination of the studies in the review, key information about participant characteristics, interventions, outcome measures, and main findings was organized in tables using Microsoft Excel spreadsheet software. This allowed for a clear presentation of each study’s results. Following the tabulation of results, a narrative synthesis was conducted, which described the variations among the studies regarding their methodologies, interventions, objectives, and outcomes.

## 3 Results

The search strategy yielded 301 potential articles, broken down as follows: 158 from PubMed, 19 from Scopus, 106 from Embase, 16 from Web of Science, and 2 identified through manual search. Twenty articles were removed due to duplication and an additional three were excluded for being written in languages other than English, leaving 278 articles for analysis. Upon reviewing the titles and abstracts, 238 articles were further excluded as they did not pertain to the study’s focus or meet the inclusion criteria. Of the remaining 40 articles, 30 were excluded for various reasons including 3 literature reviews, and 27 studies with ineligible interventions, outcome measures, or populations. - Consequently, 10 papers were selected for qualitative analysis. This entire selection and exclusion process is detailed in the PRISMA flow diagram (34) (Figure 1). Table 1 outlines the key characteristics of each study included in the review.

**Table 1:** Features of the included studies.

Authors, year	Study groups, participants	Methods	Results
Abu Alhaija et al., 2010 (19)	Group 1 consisted of 200 untreated subjects (100 males, 100 females; mean age 21.50 ± 3.35 years). Group 2 comprised 200 treated subjects (100 males, 100 females; mean age 20.92 ± 2.48 years). The subjects were all undergoing orthodontic treatment or in the retention stage of treatment.	Assessment of patients’ personality profiles and traits was carried out using the NEO-FFI. Pain and attitude toward orthodontic treatment for participants were assessed using a VAS based on a line marked at 10-mm intervals.	No notable differences were observed in any of the five personality traits describing pain perception and attitudes toward orthodontic treatment. Gender was found to be the only variable that had an effect on patients’ average pain perception (*p* < 0.01). Treated and untreated subjects had similar attitudes toward orthodontic treatment. The mean attitude score for subjects who felt pain during orthodontic treatment was 5.06 ± 1.43, whereas it was 4.32 ± 1.35 for those who did not experience pain (*p* < 0.001).
Abu Alhaija et al., 2015 (31)	A hundred subjects (50 females and 50 males). The mean ages of the included subjects were 17.5 ± 2.05 years at T1 (before treatment) and 19.15 ± 2.32 years at T2 (after treatment). Subjects were treated by fixed orthodontic appliances.	Assessment of patients’ personality profiles and traits was carried out using the NEO-FFI. Pain expectation at T1 and actual experience at T2 was assessed using a VAS. Patients’ attitude toward orthodontic treatment was assessed using a VAS marked at 10-mm intervals.	Orthodontic treatment has altered the psychological traits of patients. Scores for neuroticism decreased following treatment, while scores for openness, agreeableness, and conscientiousness increased after treatment (*p* < 0.001). The difference between the average expected pain score (T1) and the average experienced pain score (T2) was not statistically significant (*p* = 0.11). Gender differences were not detected in average pain score (*p* < 0.05). A positive attitude toward orthodontic treatment was recorded at T1 (4.31 ± 1.26) and showed improvement at T2 (3.98 ± 1.16). Significant enhancements were observed in male subjects and across the entire sample (*p* < 0.05).
Al-Nazeh et al., 2020 (37)	Fifty participants (26 females and 24 males) who were planned to be managed with Invisalign orthodontic treatment at the age of 18–48 years old (mean age = 27.62 years). Simple randomization method with gender stratification was used to select participants.	Personality attributes and characteristics were measured via NEO-FFI. Pain was assessed using a VAS based on a line marked at 10-mm intervals. Oral health impacts were measured for each participant via Oral Health Impact Profile (OHIP).	This investigation showed that personality scores had an impact on oral health outcomes before and after treatment. Hierarchical regression analysis showed among men after treatment: extraversion (*p* = 0.021), openness (*p* = 0.004) and conscientiousness (*p* = 0.005) (NEO-FFI scores) were related to OHIP results (*p* < 0.05). Other personality scores, such as neuroticism and agreeableness, did not prove useful. In women assessed with NEO-FFI, post-treatment outcomes showed no significant effect (*p* > 0.05). Among males, openness scores (*p* = 0.048) were predictive of OHIP scores before treatment.
Al-Omiri et al., 2006 (38)	Fifty patients at a mean age = 20.7 years (13–28 years, 20 males, 30 females) who were randomly selected from records of the Orthodontic Department in Irbid. Twenty-five patients were treated with the extraction of teeth, and 25 subjects were treated nonextraction. All subjects were treated with upper and lower fixed appliances.	Assessment of patients’ personality profiles and traits was carried out using the NEO-FFI. Pain assessment was associated with Dental Impact on Daily Living Questionnaire (DIDL) to satisfaction after orthodontic treatment.	There was no relationship between the level of pain (specifically, satisfaction, accepting pain) with personality traits. Borderline and high treatment need and extraction/nonextraction groups were comparable in their satisfaction with appearance, pain, general performance, and eating and chewing. Sixty-six percent of the dissatisfied and the relatively satisfied subjects had average or high neuroticism scores (*p* < 0.021). A negative correlation between neuroticism and total satisfaction was observed (*p* < 0.01). Conscientiousness was positively associated with oral comfort (*p* < 0.031).
Bergius et al., 2008 (5)	Fifty-five children: 32 females and 23 males aged 12 to 18 years (mean age 15.4 years). The patients were separated into pain and no-pain groups according to pain experiences at day 7. Elastic orthodontic separators were placed bilaterally, mesial and distal of the first molars in at least 1 jaw.	Personality factors were assessed through two questionnaires that evaluated temperament and self-concepts. Temperament was assessed with the EAS Temperament Survey for adults. Self-esteem is defined as a person’s cognitive and emotional idea about herself or himself and was tapped by using the self-report questionnaire. Pain was assessed using a VAS. Use of pain medication were also noted. Dental anxiety was assessed with the dental anxiety scale (DAS).	The study focused on factors when prolonged pain in orthodontic treatment can be predicted. The results showed significant predictive power from: low motivation for treatment, high fear of the dentist, temperament (low level of activity = low activity temperament). The pain group had significantly higher DAS values than the no-pain group.
Campos et al., 2019 (12)	Five hundred and seven subjects: (186 males-36.7% and 321 females-63.3%; age 12–60 years; mean age 26.32 ± 11.7 years). The inclusion criterion was orthodontic treatment with a fixed appliance (metallic, esthetic or self-ligating brackets), excluding rapid maxillary expansion treatment.	The personality trait assessment - MPI Orthodontic which consists of two independent parts: Part I—psychosocial aspects and Part II—behavioral aspects. Pain perception of was evaluated using a VAS and the MPI-Orthodontic.	The agreement between the classification of the pain impact level assessed by the VAS and by MPI-Orthodontic was unsatisfactory. It was noted that women (*p* = 0.009), younger individuals (*p* < 0.001), and those experiencing difficulty or pain while eating (*p* = 0.002) showed a heightened perception of both psychosocial and behavioral aspects. The model indicated that women (*p* < 0.001), younger individuals (*p* < 0.001), those from lower economic backgrounds (*p* = 0.011), individuals reporting feeding difficulties or pain (*p* < 0.001), those who did not seek treatment voluntarily (*p* = 0.013), and those dissatisfied with their treatment (*p* = 0.010) demonstrated increased psychosocial components of pain.
Cooper-Kazaz et al., 2013 (32)	Sixty-eight adult patients (23 males and 45 females), including: 28 patients with buccal appliances, 19 with lingual appliances, and 21 with clear aligners. Ages ranged from 19 to 47 years: (a) in the buccal group the mean age was 29.3 years (20–45 years); (b) lingual group the mean age was 34 years (25–47 years); (c) clear aligners group the mean age was 29.4 years (19–60 years).	The patients’ personality profile and traits were assessed: Psychological status and symptoms of mental distress was assessed by: The BSI; II. The GHSI which measure the effect of a health problem on the quality of life; III. The NVS which assess personality traits related to the existence of narcissistic personality disorder. The degree of pain was assessed using a VAS. Analgesic consumption was based on patient self-report.	Patients who reported higher levels of pain had reduced self-esteem regulation (*p* = 0.031). Patients reporting higher levels of pain had more narcissistic features (*p* = 0.005). Narcissistic vulnerability was similar across all patients and did not influence the choice of a specific orthodontic appliance. Patients selecting lingual and clear aligner appliances tended to express more somatization symptoms compared to buccal patients (*p* = 0.025).
Kadu et al., 2015 (39)	Group 1 consisted of 100 treated subjects (50 males, 50 females; average age 16.07 ± 1.36 years), and Group 2 consisted of 100 untreated subjects (50 males, 50 females; average age 16.07 ± 1.41 years).	Assessment of patients’ personality profiles and traits was carried out using the NEO-FFI. Pain and attitude toward orthodontic treatment were assessed using a VAS based on a line marked at 10-mm intervals.	Patients exhibiting high levels of trait neuroticism (*p* = 0.01) and low levels of trait conscientiousness (*p* = 0.02) reported experiencing more pain. Patients with high levels of trait conscientiousness showed better attitude (*p* = 0.01). There was a strong relationship between pain perception and attitude (*p* ≤ 0.0001). Gender and treatment status had no impact on pain perception and an individual’s attitude toward orthodontic treatment. There was no significant difference in pain perception (*p* = 0.24) or attitude toward treatment (*p* = 0.08) between the two groups.
Medonça et al., 2020 (40)	The study sample included 103 patients who had undergone orthodontic treatment with fixed appliances, comprising 40 males and 63 females (average age 20.5 years), divided into two groups: G1 (*n* = 51), which consisted of control patients who did not receive any post-procedure communication; and G2 (*n* = 52), which included patients who received a structured text message.	The patients completed a questionnaire to assess their level of anxiety during the treatment (the Modified Corah Dental Anxiety Scale-MDAS). Pain was assessed by using VAS in baseline and 10 times prospectively in predetermined time points. VAS was also applied to assess the patient’s routine alterations caused by the pain.	Low-level anxiety was observed in 42.7% of the patients, while high-level anxiety was noted in 7.8%. A statistically significant correlation between anxiety and pain was found (*p* < 0.05). At all measurement points, patients with higher levels of anxiety reported statistically significant higher pain perception scores (*p* < 0.05), except at time point T3 in Group 2 (G2), where the correlation was not significant (*p* > 0.05).
Singh et al., 2017 (30)	One hundred and fifty patients in treated group (75 males, 75 females; age 17.43 ± 1.44 years) and 150 patients in untreated group (75 males, 75 females; age 17.60 ± 1.32 years). Treated group includes who had fixed orthodontic treatment for minimum 6 months.	The personality trait assessment using the NEO-FFI. Pain perception and the attitude toward orthodontic treatment were evaluated using a VAS marking at an interval of 10 mm.	There was a statistically significant difference in pain perception between low and high levels of neuroticism (*p* = 0.009), with higher pain associated with higher levels of neuroticism. A significant difference in pain was also observed for conscientiousness across the spectrum from very low to very high levels (*p* = 0.021). A strong relationship was found between attitude and pain perception (*p* = 0.001). Additionally, there was a strong correlation between attitude and conscientiousness (*p* = 0.01), which was directly proportional. However, the mean pain perception (*p* = 0.26) and the mean values of attitude (*p* = 0.09) did not show statistically significant differences between the groups. Attitude also did not show a statistically significant difference between males and females in both the untreated (*p* = 0.49) and treated groups (*p* = 0.58).

NEO-FFI, NEO Five-Factor Inventory [NEO refers to neuroticism (N), extraversion (E), and openness (O)]; VAS, visual analog-scale; BSI, Brief Symptom Inventory; GHSI, Glasgow Health Status Inventory; NVS, Narcissistic Vulnerability Scale; EAS, Emotionality, Activity and Sociability Temperament Survey.

### 3.1 Results of the quality assessment

The quality assessment results for each study are summarized in Table 2. Using the NOS assessment (36), five studies were evaluated as having a low risk of bias (12, 19, 32, 37, 38), while another five studies were considered to have a moderate risk of bias (5, 30, 31, 39, 40). There was generally high variability observed across the study designs, objectives, populations, and evaluation methods.

**Table 2:** The quality assessment of the studies included.

The quality assessment of the non-randomized studies (NOS)				
Authors, year	Selection	Comparability	Outcome	Total score
Abu Alhaija et al., 2010 (19)	***	**	**	7
Abi Alhaija et al., 2015 (31)	**	**	**	6
Al-Nazeh et al., 2020 (37)	***	**	**	7
Al-Omiri et al., 2006 (38)	***	**	**	7
Bergius et al., 2008 (5)	**	**	**	6
Campos et al., 2019 (12)	**	**	**	7
Cooper-Kazaz et al., 2013 (32)	***	**	**	7
Kadu et al.,2015 (39)	**	**	**	6
Medonça et al., 2020 (40)	**	**	**	6
Singh et al., 2017 (30)	**	**	**	6

NOS-Newcastle–Ottawa Quality Assessment Scale. In the NOS assessment a study can be awarded a maximum of one star for each numbered item within the selection and outcome categories. A maximum of two stars can be given for comparability. [*] are assigned to three criteria, i.e., selection (with a maximum of 4 stars [****]), comparability (with a maximum of 2 stars [**]), and outcome (with a maximum of 3 stars [***]). The total score was attributed to the categories of “high risk of bias” (total score of 0–3), “moderate risk of bias” (total score of 4–6), and “low risk of bias” (total score of 7–9).

### 3.2 Characteristics of the study groups (age, gender, orthodontic method/appliances)

The studies encompassed a total of 1,833 patients of varying ages. Three of the included articles concentrated on exploring the relationship between personality traits and pain perception during orthodontic treatment in children and adolescents (5, 30, 39). The remaining seven studies included adults (12, 19, 31, 32, 37, 38, 40).

When looking at individual studies and considering the gender of participants, it is noticeable that women were more commonly involved. Women accounted for 60% in the study of Al-Omiri et al. (38), 61.2% in the study of Medonça et al. (40), and 63.3% in the study of Campos et al. (12). A similar percentage of women was observed in the research of Cooper-Kazaz et al. (32) (66.2%). The study of Bergius et al. (5) involved 32 women, which constitutes 58.2% of the respondents. In the study by Al-Nazeh et al. (37) women accounted for 52% of the study participants. Perfect match in terms of gender of the subjects was noted in the studies by Singh et al. (30) (75 women and 75 men in both the treated and untreated groups), Abu Alhaija et al. (19) (100 women and 100 men in both groups), as well as Abu Alhaija et al. (31) and Kadu et al. (39) (50 women and 50 men in both groups).

Three authors conducted studies comparing untreated and orthodontically treated groups (19, 30, 39). In the study by Abu Alhaija et al. (19), the treated group consisted of patients currently undergoing orthodontic treatment or in the retention phase, while the untreated group had no prior orthodontic treatment. Singh et al. (30) included in the treated group patients who had undergone fixed orthodontic treatment for at least 6 months. Kadu et al. (39) focused on patients who had either completed their orthodontic treatment or were at least 6 months into their scheduled treatment. Other researchers focused solely on the treated group. Abu Alhaija et al. (31) assessed personality traits, attitudes toward orthodontic treatment, and pain perception/experience before and after treatment with fixed appliances. Campos et al. (12) included patients undergoing treatment with various fixed appliances, while Medonça et al. (40) studied those with fixed appliances. Al-Omiri et al. (38) compared patients treated with fixed appliances, differentiating between those who had teeth extracted and those who did not. Cooper-Kazaz et al. (32) categorized participants based on the type of orthodontic appliances used: buccal, lingual, or clear aligners. In the study by Al-Nazeh et al. (37), all participants were treated with Invisalign. Lastly, Bergius et al. (5) examined pain perception and psychological traits in subjects using flexible orthodontic spacers.

### 3.3 Association between personality traits and pain perception during orthodontic treatment

In most of studies the assessment of pain sensation (5, 12, 19, 30–32, 37, 39, 40), as well as attitude toward orthodontic treatment (19, 30, 31, 39) was evaluated using a visual-analog scale (VAS) marked at 10-mm intervals and the assessment of personality model was carried out using the NEO Five Factor Inventory (NEO-FFI) - NEO refers to neuroticism (N), extraversion (E), and openness (O) (19, 30, 31, 37–39). In addition, Campos et al. (12) assessed the psychosocial and behavioral aspects of pain perception using the Multidimensional Pain Inventory (MPI) which was adapted to orthodontic patients (MPI-Orthodontic) to assess pain perception and investigate the impact of pain on the lives of orthodontic patients. Al-Omiri et al. (38) used the Dental Impact on Daily Living (DIDL) questionnaire to assess the effect of orthodontic treatment on daily living and satisfaction with the dentition, namely appearance, pain, oral comfort, general performance, and chewing and eating. In the study of Cooper-Kazaz et al. (32) patients completed a daily Health-Related Quality of Life (HRQOL) questionnaire. Patients’ perceptions of pain intensity and dysfunction were measured in four areas. Pain intensity was also checked by analgesic consumption analysis. These authors used tools to assess patients’ personality traits as follows: the Brief Symptom Inventory (BSI) was used to evaluate psychological status and symptoms of mental distress, the Glasgow Health Status Inventory (GHSI) assessed the impact of health issues on quality of life, and the Narcissistic Vulnerability Scale (NVS) measured personality traits associated with narcissistic personality disorder (32). Similarly to Cooper-Kazaz et al. (32), Bergius et al. (5) noted, in addition to the VAS scale, the use of pain medication to assess the perception of pain by subjects. Bergius et al. (5) evaluated the personality factors by using two questionnaires assessing temperament and self-concepts. Temperament was assessed with the EAS (Emotionality, Activity and Sociability) Temperament Survey for adults. Self-esteem was defined as a person’s cognitive and emotional idea about herself or himself and was tapped by using the self-report questionnaire. In addition, Al-Nazeh et al. (37) assessed what factors (e.g., psychological traits) were associated with the Oral Health Impact Profile (OHIP) value. OHIP determines the impact on oral health and quality of life, including dysfunction, discomfort and disability. Medonça et al. (40) used a questionnaire to assess the patients’ level of anxiety during the treatment (the Modified Corah Dental Anxiety Scale-MDAS).

The main findings on the association between different personality traits and pain perception are summarized in Table 3. Both Singh et al. (30) and Kadu et al. (39) established a robust link between pain perception and attitude toward orthodontic treatment. Their findings indicated that a more positive attitude led to decreased pain perception. Additionally, the authors found a strong correlation between pain perception and personality traits, such as neuroticism and conscientiousness that is more pain with higher levels of neuroticism and lower conscientiousness levels. On the other hand, the attitude toward treatment was significantly correlated with conscientiousness, with higher levels of conscientiousness associated with a more positive attitude. Patients who experienced less pain during orthodontic treatment tended to have a more positive attitude. The average pain perception and attitudes were comparable between treated and untreated groups, and pain was not influenced by gender. However, in a similar research setup, Abu Alhaija et al. (19) observed no significant differences across any of the five personality traits in relation to attitude toward orthodontic treatment and pain perception. In contrast, they noted that gender was the sole factor affecting average pain perception, with females being more sensitive to pain than males. In both treated and untreated groups, attitudes toward orthodontic treatment were alike, with a more positive attitude noted among patients who experienced less pain. Conversely, Al-Omiri et al. (38) reported no association between pain levels and personality traits. However, neuroticism influenced overall treatment satisfaction. Dissatisfied and relatively satisfied subjects had average or high neuroticism scores and none of them demonstrated a low neuroticism score.

**Table 3:** The summary of the main findings on the association between different personality traits and pain perception.

Authors, year	Association between different personality traits and pain perception
Abu Alhaija et al., 2010 (19)	Personality traits had no impact on patients’ average pain perception.
Abu Alhaija et al., 2015 (31)	Neuroticism scores were reduced after orthodontic treatment. Openness, agreeableness and conscientiousness scores increased after treatment.
Al-Nazeh et al., 2020 (37)	Extraversion, openness and conscientiousness scores were useful among males after orthodontic treatment to predict OHIP scores.
Al-Omiri et al., 2006 (38)	There were no association between pain levels and personality traits. Neuroticism influenced overall treatment satisfaction.
Bergius et al., 2008 (5)	Lower motivation was associated with higher levels of pain. Higher pain levels were found in the high-dental anxiety scale group and in the low-activity temperament group.
Campos et al., 2019 (12)	Subject who did not seek treatment voluntarily and who were not satisfied with the treatment, exhibited greater psychosocial components of pain.
Cooper-Kazaz et al., 2013 (32)	Patients who reported higher levels of pain had reduced self-esteem regulation and more narcissistic features.
Kadu et al., 2015 (39)	Personality traits, such as neuroticism and conscientiousness were associated with pain perception. Increased pain perception was associated with higher levels of neuroticism and lower levels of conscientiousness.
Medonça et al., 2020 (40)	Patients with higher levels of anxiety reported statistically significant higher pain perception scores.
Singh et al., 2017 (30)	Neuroticism and conscientiousness were associated with pain perception. Increased pain perception was associated with higher levels of neuroticism and lower levels of conscientiousness.

OHIP, Oral Health Impact Profile.

Cooper-Kazar et al. (32) found that patients who reported higher pain levels also had lower self-esteem regulation and more pronounced narcissistic traits. The study also explored personality and psychological characteristics to understand how they might influence patients’ preferences for different types of braces. They noted that narcissistic vulnerability did not affect the choice of a specific orthodontic appliance. Bergius et al. (5) reported that pain perception during orthodontic treatment was linked to motivation, with lower motivation associated with higher levels of pain. Higher pain levels were also found in the high-dental anxiety scale group and in the low-activity temperament group. Similarly, Medonça et al. (40) showed the significant correlation between anxiety and pain with higher scores for pain perception in patients with higher levels of anxiety. Abu Alhaija et al. (31) showed changes of the psychological characteristics of patients after orthodontic treatment. Neuroticism scores were reduced after orthodontic treatment. On the other hand, openness, agreeableness and conscientiousness scores increased after treatment. Gender differences were not detected in average pain and attitude score. The average positive attitude toward orthodontic treatment improved after orthodontic treatment. Al-Nazeh et al. (37) showed that only among males after orthodontic treatment with Invisalign to predict OHIP scores extraversion, openness and conscientiousness scores were useful, meanwhile, other personality scores (neuroticism and agreeableness) had no effect. Capmos et al. (12) found that subject who did not seek treatment voluntarily and who were not satisfied with the treatment, exhibited greater psychosocial components of pain.

## 4 Discussion

While orthodontists implement various techniques to reduce discomfort, patients often still experience pain during treatment. Pain is an unpleasant sensory and emotional response linked to actual or potential tissue damage and can be a significant deterrent to undergoing orthodontic treatment. Interestingly, even with consistent stimuli like the initial placement of archwires, pain perception varies widely among individuals (10, 41, 42). This variation can be attributed to both general and personal factors such as motivation, gender, past negative dental experiences, and dental phobia (39). The reason why some people are more susceptible to pain induced by orthodontic procedures continues to be explored. The influence of a patient’s psychological traits and personality on pain perception during treatment has been highlighted (29, 43). Moreover, the success of orthodontic treatment largely depends on the patient’s cooperation and motivation. Understanding the personalities of orthodontic patients can lead to enhanced patient satisfaction, more successful treatments, and improved oral health (13, 17, 39).

This review provides a detailed analysis of the connections between patients’ personality traits and pain perception during orthodontic treatment. It includes 10 studies that explore the personality profiles and pain experiences of patients undergoing treatment with different orthodontic methods and appliances. The majority of these studies found correlations between personality traits and factors like pain perception, treatment attitudes, and patient satisfaction (5, 12, 30–32, 37–40). However, the findings of some studies remain inconclusive (19). Five of these studies were considered to have a low risk of bias (12, 19, 32, 37, 38). It is important to note the significant heterogeneity observed among these studies in terms of design, objectives, populations studied, treatment types, timing of personality assessments, and the tools used for evaluating pain perception and personality. Most studies involved adult participants (12, 19, 31, 32, 37, 38, 40), with a higher proportion of female subjects (5, 12, 32, 37, 38, 40). Pain perception was commonly assessed using a Visual Analog Scale (VAS) at 10 mm intervals, offering outcomes ranging from extremely likely to extremely unlikely, proving to be a reliable and sensitive measure (5, 12, 19, 30–32, 37, 39, 40). Personality traits were often evaluated using the NEO-FFI test (19, 30, 31, 37–39), which effectively measures the five major personality aspects: neuroticism, extraversion, openness, agreeableness, and conscientiousness. This test is recognized for its brevity, reliability, comprehensiveness, and validity in assessing an individual’s personality traits (19, 38, 44).

The available literature demonstrated a relationship between neuroticism and conscientiousness with pain sensation during orthodontic treatment (30, 31, 39). According to Singh et al. (30) and Kadu et al. (39), lower levels of conscientiousness were associated with increased pain perception, while higher levels of neuroticism correlated with higher degrees of pain perception. From a clinical point of view this is crucial information for orthodontists, as patients with high neuroticism may benefit from simultaneous psychological support throughout their treatment. On the other hand, some personality traits, such as neuroticism may significantly impact on the cooperation during orthodontic treatment with removable appliances, including clear removable aligners. Thus, personality traits can affect the choice of the type of orthodontic therapy. Additionally, neuroticism was reported to affect post-treatment satisfaction. In the study by Al-Omiri et al. (38), patients with higher neuroticism scores exhibited lower satisfaction with their teeth after orthodontic treatment. This finding aligns with Kiyak et al. (45), who noted that patients with higher neuroticism scores were less satisfied immediately after surgery but expressed increased satisfaction later. On the other hand, Abu Alhaija et al. (31) observed that after orthodontic treatment, neuroticism scores decreased while scores for openness, agreeableness, and conscientiousness increased, leading to an improved attitude toward orthodontic treatment. These findings are consistent with those of Varela and García (43) and Cunningham et al. (46), who reported improvements in emotional stability and increased self-confidence following orthodontic treatment. These changes in personality scores may be linked to a reduction in dental fear as patients become more familiar with their orthodontist and orthodontic appliances. Similarly, Bos et al. (25) reported an increased positive attitude in treated patients compared to untreated ones, which can be attributed to personal knowledge and information gained from orthodontic experiences. However, these results differ from those of Abu Alhaija et al. (19) and Lagerström et al. (47), who found no significant difference in attitudes toward orthodontic treatment between treated and untreated patients.

The relationship between personality traits, pain perception, post-treatment satisfaction, and patient motivation is also significant. Campos et al. (12) found that individuals dissatisfied with their treatment exhibited more pronounced psychosocial aspects of pain, which is closely related to their level of motivation. Motivation, defined as the willingness to make efforts to achieve a goal, is crucial in orthodontic treatment. In many studies, motivation is described in terms of the reason or desire for treatment (5). A lack of motivation can lead to additional difficulties, not only with the treatment protocol itself but also with the patient’s overall management and response to therapy (38). Bergius et al. (5) also demonstrated a link between motivation and pain, showing that low motivation in orthodontically treated patients could predict the occurrence of pain during therapy. Furthermore, Bergius et al. (5) identified other predictive factors, noting that a temperamental trait characterized by low activity levels and high dental fear were significant indicators of high pain levels. In a review of persistent pain, Keefe et al. (48) highlighted psychological factors associated with poor adjustment to pain, including pain catastrophizing, pain-related anxiety and fear, and helplessness. Patients scoring high on helplessness reported higher levels of pain, depression (including low activity), disability, and significantly poorer treatment outcomes. Similarly, Medonça et al. (40) found a correlation between anxiety and pain, with higher pain perception scores observed in patients with higher levels of anxiety. These arrangements were in line with those of White et al. (16), who reported a positive association between dental anxiety and patient’s pain perception during orthodontic treatment.

Cooper-Kazaz et al. (32) explored whether personality and psychological characteristics influence patients’ preferences for orthodontic appliances. They found that a patient’s choice of appliance might reflect their personality traits or psychological status, affecting their adaptation and adjustment to the appliances. Interestingly, narcissistic vulnerability did not influence appliance selection, despite expectations that individuals with these traits might prefer less noticeable appliances. Patients who opted for lingual braces and clear aligners reported more somatization symptoms compared to those with buccal appliances, with the lingual group also exhibiting more obsessive-compulsive symptoms. While narcissistic traits did not affect the choice of braces, they did impact pain coping. Patients who reported higher pain levels had poorer self-esteem regulation and exhibited more narcissistic features. These findings differ from those of Abu Alhaija et al. (19), who found no significant correlation between personality traits and pain perception or attitude toward orthodontic treatment. Similarly, Bos et al. (28) concluded that personality traits alone cannot predict patient cooperation during orthodontic treatment. Amado and Sierra (49) also reported no significant associations between psychological characteristics and cooperation. However, Abu Alhaija et al. (19) noted that patients with a more positive attitude experienced less pain during orthodontic treatment. In their study, pain perception was lower in patients with prior knowledge about orthodontic treatment, consistent with findings by Touyz and Marchand (50), who suggested that informing patients about expected discomfort can reduce pain during treatment. Additionally, Abu Alhaija et al. (19) found that while personality traits did not affect pain perception, gender did, with females showing greater pain sensitivity than males, aligning with previous research (49, 51). In this context, it is important to mention that many other factors can affect pain perception during orthodontic treatment, including patient’s age and the type of orthodontic methods or appliances used. There appear to be conflicting findings with regards to age differences in orthodontic pain experience, which may be due to various treatment approaches. Some authors observed that adolescents report higher intensity of pain after orthodontic appliance activation than preadolescents and adults (6, 8). On the other hand, several studies reported that the older the patient, the greater the pain reported, and the greater the pain sensitivity, and the lower the pain tolerance (7, 52). Regarding orthodontic pain associated with fixed appliances and clear aligners, it has been proved that during the first week of orthodontic treatment, patients treated with clear aligners reported lower pain than those treated with fixed conventional and self-ligating appliances (53, 54). It should also be noted that pain is one of the factors that may affect sleep quality and perceived life satisfaction. As patients experiencing pain demonstrated poor sleep quality and associated reduced life satisfaction, this is an important issue with strong clinical and socioeconomic implications (55).

It is also important to recognize that personality factors such as extraversion, openness, and conscientiousness influence oral health-related quality of life (OHIP scores) (37). In this context, it should be pointed out that OHIP should be validated to different population groups. Extraversion is linked to a greater eagerness to recognize changes in oral conditions and their effects on oral health. Openness may be associated with a greater willingness to express opinions, concerns, and attitudes toward changes in oral health. Conscientiousness is likely related to a higher degree of commitment, organization, and reporting of changes in oral status. This aligns with prior research that connects personality traits with oral health-related quality of life in various orthodontic treatments (37, 56, 57). Higher levels of extraversion and openness were associated with a lesser impact of orthodontic treatment needs on oral health-related quality of life (57). In the study by Al-Nazeh et al. (37), females had lower OHIP scores after treatment compared to baseline, while males showed no difference between baseline and post-treatment OHIP scores. These results are consistent with previous studies indicating that females are more likely to accept and be satisfied with orthodontic therapy (58), and tend to prioritize their dental and oral health more than males (38, 59). However, this contrasts with other studies that found no relationship between gender and satisfaction with orthodontic treatment (19, 28, 31, 38, 49). This discrepancy could be attributed to differences in the types of orthodontic treatment used, racial factors, psychosocial considerations, timing of assessments during the study, and/or study design.

This systematic review presents several limitations that should be acknowledged: (a) only five studies were assessed as having a low risk of bias based on the NOS tool; (b) there was high variability in study designs and aims, characteristics of study groups, and study methods, including differences in the timing of personality assessments and the use of various tools to evaluate pain perception and personality; (c) differences in the applied treatments/types of interventions (orthodontic methods/appliances), may influence the results; (d) many other factors, such as racial backgrounds, cultural, social, and demographic factors, may also impact the results of the included studies; (e) only articles in English were included in this review. Consequently, considering these limitations, further research is needed to strengthen the evidence on this topic.

## 5 Conclusion

In summary, patients’ psychological characteristics appear to influence pain perception and other factors associated with orthodontic treatment, including treatment attitudes, and post-treatment satisfaction. Understanding the relationship between a patient’s personality traits and pain experience during orthodontic treatment can enhance patient satisfaction, leading to more successful orthodontic outcomes and improved oral health. These findings are crucial from a clinical point of view, as patients with some specific personality traits may need simultaneous psychological support throughout their treatment. In addition, personality characteristics can affect the patient’s cooperation during treatment and thus may have an impact on the choice of the type of orthodontic therapy. However, given that several studies were assessed to have a moderate risk of bias and displayed significant variability in design, types of interventions, study populations, and methods, there is a need for larger, high-quality studies with consistent design and methodology to confirm the findings of this review.

## Data Availability

The original contributions presented in the study are included in the article/supplementary material, further inquiries can be directed to the corresponding author.
